# A Case of Pediatric Tracheal Stenosis Secondary to Chronic Retching in the Setting of Bulimia Nervosa

**DOI:** 10.1155/crpe/9967394

**Published:** 2025-02-22

**Authors:** Arpan Patel, Charles Saadeh

**Affiliations:** ^1^Texas College of Osteopathic Medicine, University of North Texas Health Science Center, Fort Worth, Texas, USA; ^2^Department of Otolaryngology, Cook Children's Medical Center, Fort Worth, Texas, USA

## Abstract

This report presents a case of acquired tracheal stenosis in a pediatric patient without a history of prolonged intubation. A 14-year-old female presented with a chief complaint of biphasic stridor and a medical history remarkable for bulimia nervosa and one year of self-induced vomiting. Endoscopic evaluation revealed Grade three tracheal stenosis. Lab work was unrevealing for acute inflammatory process nor vasculitis. Pathology was unremarkable. The patient tolerated primary dilation and second look did not reveal further stenosis. Her extensive workup failed to reveal an alternative etiology with a working hypothesis that the stenosis was a result of chronic retching due to bulimia.

## 1. Introduction

Tracheal stenosis is an important life threatening pathology in the pediatric population. The prompt diagnosis and management of tracheal stenosis is crucial to prevent complications of airway obstruction. Common etiologies include congenital anomalies, traumatic injury, complications of infection, and autoimmune disorders [1]. Diagnosis is made with a combination of cross sectional imaging and rigid bronchoscopy [2]. Endoscopic assessment and intervention will generally relieve acquired tracheal stenosis, with more severe cases requiring open surgical approaches [3]. Historically, pediatric tracheal stenosis has had a poor prognosis; however, notable advances in endoscopic and open surgical treatments have led to a decline in morbidity and mortality [1].

This case report highlights a unique presentation of acquired tracheal stenosis in a 14-year-old female without a history of prolonged intubation and normal inflammatory markers and autoimmune workup. Ultimately, her etiology was attributed to bulimia nervosa due to prolonged self-induced vomiting. Eating disorders, particularly bulimia, are increasingly recognized for its effect on the aerodigestive tract [4–7]. Although a rare presentation in pediatric patients, tracheal stenosis due to chronic retching serves as an example of the intricate connection between behavioral health disorders and their direct or indirect physical ramifications [8].

## 2. Case Report

**Figure 1:**
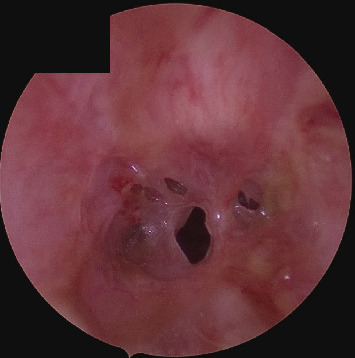
Preoperative endoscopy showing Grade 3 tracheal stenosis.

A 14-year-old female presented to the Emergency Department at Cook Children's Medical Center with biphasic stridor and evidence of airway obstruction. The patient had never been tracheally intubated and had no other significant medical history except for 1 year of frequent retching in the setting of bulimia nervosa. Symptoms were gradual in onset over the course of several weeks, with an initial working diagnosis of asthma with no improvement with inhaled steroids. A CT scan was obtained that revealed severe tracheal stenosis involving the cervical trachea. Initial lab work revealed an elevated WBC of 16.7, potassium of 2.8 mmol/L, normal C-reactive protein, erythrocyte sedimentation rate, and procalcitonin. Her initial assessment was concerning for impending airway obstruction and she was taken to the operating room for emergent rigid bronchoscopy. She was found to have an irregular circumferential scar extending for 1.5 cm, starting 2 cm distal to the glottis involving the second through fourth tracheal rings (Figure 1). The initial stenosis was grade three with pinpoint 2-3 mm patency. The scar was biopsied, and the stenosis easily dilated to normal caliber. Circumferential submucosal Kenalog was applied and the patient ultimately did very well clinically and was discharged on postoperative day 1 in stable condition.

Her workup included antineutrophil cytoplastic antibodies, myeloperoxidase antibodies, and serine proteinase 3 antibodies, which were all normal. Her pathology revealed chronic tracheitis without evidence of bacterial tracheitis or vasculitis. Intraoperative cultures were unremarkable.

**Figure 2:**
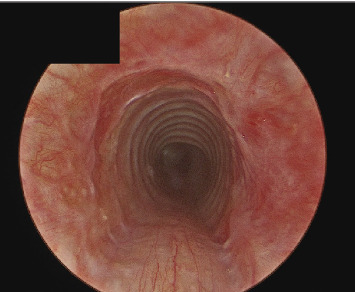
6 weeks' postoperative endoscopy showing healthy tracheal lumen.

She was taken back to the operating room 6 weeks later with findings of a normal lumen trachea with no evidence of restenosis or granulation (Figure 2). Of note, she had refrained from further retching since her initial presentation.

At 1 year postoperative, the patient developed restenosis to the same degree as initially observed. A workup for IgG4-related disease was considered, as staining showed scant positivity, though it was insufficient for a definitive diagnosis. Comprehensive laboratory evaluation, including inflammatory markers and autoimmune panels, was unremarkable. Despite unclear postoperative purging behavior, she continued to exhibit self-harming tendencies. Given the potential for IgG4-related disease, she was initiated on immunosuppressive therapy with Remicade, per the recommendation of the rheumatology division. Currently, she is responding well to treatment. The differential diagnosis remains narrowed to idiopathic, traumatic (secondary to bulimia), or unlikely IgG4-related disease.

## 3. Discussion

Pediatric tracheal stenosis is relatively rare, and the majority of cases can be attributed to etiologies such as congenital anomalies, trauma, complications of infection, and autoimmune disorders [1]. This patient's presentation was unique in that she had none of these risk factors and the only part of her history that contributed to aerodigestive trauma was chronic retching in the setting of bulimia. While not definitive, her intraoperative pathology did not suggest vasculitis, and in context of normal inflammatory markers and normal antibody panel for vasculitis, we were able to rule this out with the help of our rheumatology colleagues.

This particular case deviates from the conventional presentations of tracheal stenosis, while also shedding light on the potential physical consequences of behavioral health disorders.

Patients with eating disorders, such as bulimia nervosa, may engage in behaviors like inserting fingers into their throat to stimulate the pharynx or applying pressure to the abdomen to induce vomiting [9]. The interplay of digital trauma to the supraglottis with local trauma from frequent retching can lead to locoregional trauma that can contribute to tracheal scar. More importantly, there is potential for chronic aspiration of caustic stomach contents that further contribute to tracheal stenosis [10]. Interestingly, despite the possibility of chronic aspiration of gastric contents, there was no evidence of aspiration pneumonia in her clinical course or imaging. The complex interplay of mechanical pressure, chemical irritants, and chronic exposure to these factors likely contributed to the formation of the acquired tracheal stenosis observed in this patient [11]. The pathophysiology of tracheal stenosis often involves mechanical irritation and pressure on the tracheal wall, which can lead to mucosal damage, mucosal necrosis, and eventually fibrous granuloma or scarring in the tracheal mucosa [12]. In cases of prolonged tracheal intubation, the mechanical pressure exerted on the tracheal wall has been identified as a significant factor in stenosis formation [2]. Similarly, the constant irritation and pressure on the trachea resulting from self-induced vomiting can lead to similar tracheal irritation and stenosis, as observed in our patient. The endoscopic image of the stenosis, with its resemblance to decubitus lesions caused by tracheotomy cannulas, raises the question of whether the patient could have used a blunt object to stimulate vomiting, although no evidence or history of such behavior was elicited during evaluation. It is worth noting that patients often conceal these behaviors, making it challenging to elicit a comprehensive medical history [8].

Bulimia nervosa, characterized by recurrent binge eating followed by purging, manifests a spectrum of aerodigestive complications beyond tracheal stenosis. The repetitive, self-induced vomiting can induce esophageal stenosis, laryngeal and glottic trauma, enlarged gastric capacity, dental complications, and gastroesophageal reflux disease [13].

In the differential diagnoses for the presented case, idiopathic subglottic stenosis (ISS) must be considered. However, ISS typically arises in middle-aged women and the patient was 14 years old. Second, the patient's tracheal stenosis did not have subglottic involvement [14, 15]. The characteristics of this patient's stenosis were not consistent with the classic presentation of ISS; however, there may be a similar pathophysiology as GERD is suspected to be a potential cause of ISS [15]. Another point of interest is that the stenosis involved only a short segment of the tracheal anatomy, specifically the second through fourth tracheal rings. This localized involvement contrasts with the diffuse compromise of multiple rings often seen in inflammatory or infiltrative tracheal conditions.

## 4. Conclusion

In conclusion, this case emphasizes the diverse etiologies of tracheal stenosis and, specifically, the potential development of acquired tracheal stenosis related to self-induced retching in patients with eating disorders. Furthermore, this case underscores the need to consider the systemic effects of mental health disorders in pediatric patients and helps add to our fund of knowledge regarding aerodigestive complications of bulimia nervosa.

## Data Availability

Data sharing is not applicable to this article as no datasets were generated or analyzed during the study.
